# Low-Risk and High-Risk NSMPs: A Prognostic Subclassification of No Specific Molecular Profile Subtype of Endometrial Carcinomas

**DOI:** 10.3390/cancers16183221

**Published:** 2024-09-21

**Authors:** Matteo Marchetti, Giulia Spagnol, Tommaso Vezzaro, Sofia Bigardi, Orazio De Tommasi, Emma Facchetti, Marta Tripepi, Diletta Costeniero, Chiara Munerol, Tiziano Maggino, Donato D’Antona, Roberto Tozzi, Carlo Saccardi, Marco Noventa

**Affiliations:** Unit of Gynecology and Obstetrics, Department of Women and Children’s Health, University of Padua, 35100 Padua, Italy; giuliaspagnol.ts@gmail.com (G.S.); tiziano.maggino@gmail.com (T.M.); donato.dantona@unipd.it (D.D.); roberto.tozzi@unipd.it (R.T.); carlo.saccardi@unipd.it (C.S.); marco.noventa.2@unipd.it (M.N.)

**Keywords:** endometrial cancer, molecular classification, no specific molecular profile, estrogen receptor, prognosis, risk class, NSMP

## Abstract

**Simple Summary:**

The no specific molecular profile (NSMP) subtype of endometrial cancers remains a poorly understood class from a molecular standpoint, with a wide range of prognoses due to their internal heterogeneity. In this article, we confirm the value of a previously proposed further subdivision of NSMP into two risk classes: low-risk and high-risk; they are characterized by marked differences in oncological outcomes, including both the risk of recurrence and mortality. We are confident that this subdivision could bring significant benefits in the near future, not only in terms of modulating adjuvant therapy but also in standardizing research cohorts for more consistent and reproducible data.

**Abstract:**

(1) Background: Endometrial carcinoma (EC) classified as no specific molecular profile (NSMP) represents a heterogeneous group with variable prognoses. This retrospective, single-center study aims to further stratify NSMP ECs to tailor treatment strategies and improve outcomes. (2) Methods: From 2020 to 2023, we collected data on 51 patients diagnosed with NSMP EC following the introduction of molecular profiling at our institution. Patients were retrospectively analyzed for estrogen receptor (ER) status, histotype, and grade to identify potential prognostic subgroups. (3) Results: Our analysis identified two distinct subgroups within NSMP EC: low-risk and high-risk, based on ER status, histotype, and grade. The low-risk NSMP group demonstrated significantly better survival outcomes compared to the high-risk group. With a median follow-up time of 16 moths (IQR 13.0–29.7), the disease-free survival (DFS) and overall survival (OS) for the low-risk group were 100%. For the high-risk group, the DFS and OS were 71.4% and 78.6%, respectively, which showed a statistically significantly difference (Log-Rank Mantel-Cox < 0.001). In the high-risk group, four patients experienced recurrence, and three of these patients died. (4) Conclusions: Stratifying NSMP EC into low-risk and high-risk categories based on ER status, histotype, and grade can lead to more accurate prognostic assessments. In time, it may require tailored adjuvant therapies and a personalized treatment.

## 1. Introduction

Endometrial cancer (EC) is a heterogeneous disease with variable prognostic outcomes. Traditionally, histopathological evaluation has been the cornerstone of EC classification, which involves histotype, grade, stage, myometrial invasion and lymph vascular space invasion (LVSI) to provide prognostic information and guide treatment decisions. However, patients with similar EC profiles can exhibit different outcomes, particularly in high-risk cases.

Recent advancements in molecular pathology have identified four distinct molecular subgroups of EC, associated with different prognosis: POLE ultramutated (*POL*Emut), mismatch repair-deficient (MMRd), p53 mutant (p53abn), and no specific molecular profile (NSMP) [1,2,3,4,5,6]. This classification emerged from The Cancer Genome Atlas (TCGA) project [7] and was adapted to clinical practice through the Proactive Molecular risk classifier for Endometrial Cancer (ProMisE) trial, which provided a comprehensive molecular characterization of EC and highlighted the heterogeneity within histologically defined categories [4,5,6]. Among these molecular classifications, NSMP tumors represent a significant (~50%) yet less well-defined subset that demands further elucidation. They present none of the molecular features seen in the other three subgroups, making their management particularly challenging and justifying the heterogeneity [4]. Histologically, NSMP tumors often resemble endometrioid carcinoma but lack the high mutational burden or specific genetic alterations typical of *POLE*mut [8] and MMRd tumors. Unlike p53abn tumors, which are associated with poor prognosis [9], NSMP tumors do not show the aggressive behavior associated with *TP53* mutations. The clinical behavior of NSMP tumors can be variable, with outcomes that are generally intermediate between the favorable prognosis of *POLE*mut tumors and the poorer outcomes associated with p53abn tumors.

One of the primary challenges in managing NSMP tumors is the lack of specific molecular targets for therapy. While *POLE*mut and MMRd tumors have shown responsiveness to immunotherapy due to their high mutational burden and associated neoantigen display [10,11], NSMP tumors do not exhibit these features, limiting the applicability of such treatments [12]. To date, no specific biomarker has been identified in these tumors; therefore, current research is focused on identifying novel biomarkers and therapeutic targets specific to NSMP tumors [13,14,15,16].

Understanding NSMP tumors is also essential to refine and customize adjuvant treatment and consider de-escalation strategies [12,17]. Current treatment protocols for EC are still largely based on histopathological criteria. However, the evaluation of molecular classification is increasingly encouraged for all ECs, particularly for high-risk tumors [3,18]. Also, a new risk stratification system that incorporates molecular classification has been introduced in the latest European treatment guidelines [18,19]. For instance, the ongoing PORTEC4a trial [20] aims to determine the added value of integrating molecular parameters into adjuvant treatment decisions, potentially refining the approach for NSMPs and other ECs subgroups. With the integration of molecular features into EC management, clinicians can develop more personalized treatment plans that account for the unique biological behavior of each tumor subtype. This approach has the potential to increase prognostic accuracy and enhance therapeutic efficacy and reduce overtreatment, particularly for patients with less aggressive disease. Such an approach was endorsed by the FIGO (International Federation of Gynecology and Obstetrics) with the new 2023 classification, by incorporating molecular characteristics [21].

As molecular diagnostics continue to evolve, the precise classification and targeted management of NSMP tumors will be essential for improving patient outcomes and advancing the field of gynecologic oncology. A preliminary prognostic subdivision of NSMP tumors was suggested by Vermij et al. in 2023, testing the expression of the estrogen receptor (ER) [22]. Recently, a large multicenter Canadian study proposed a subclassification of NSMP into low-risk NSMP and high-risk NSMP based on ER status and grade, resulting in two extremely different populations in terms of disease-free survival (DFS) and disease-specific deaths (DSD) [23]. This finding highlights the need to tailor adjuvant therapy accordingly.

In this study, we aimed to investigate the division of NSMP ECs into two subgroups with different prognoses, namely low-risk and high-risk NSMP, based on ER status, grade and histotype.

## 2. Materials and Methods

### 2.1. Study Design and Population

This study was designed as a retrospective analysis of a prospectively maintained single-center database, including women with EC diagnosed at any stage. We selected patients with NSMP EC who underwent primary surgical resection ± adjuvant treatment at the Gynecological Department of the University Hospital of Padua, Italy, between April 2020 and December 2023. This study aimed to identify prognostic factors that help define subgroups of patients with NSMP endometrial carcinoma characterized by different prognoses with the goal of more accurately tailoring adjuvant therapy towards treatment de-escalation. During the study period, a systematic molecular investigation was conducted for all resected endometrial specimens according to ProMise classification and ESGO (European Society of Gynaecological Oncology) 2020 guidelines [19]. This study was approved by the ethics committee of the University Hospitals of Padua with the Protocol Number: 5696/AO/23. Patients with an ‘ER-positive/endometrioid/low-grade’ phenotype formed the low-risk group. All other tumors were considered high-risk NSMPs.

The inclusion criteria were as follows: a diagnosis of primary EC of any histology; no prior neoadjuvant radiation or chemotherapy; completion of surgical staging, including at a minimum, hysterectomy and bilateral salpingo-oophorectomy; availability of sufficient tissue (from hysteroscopic biopsy, hysterectomy, or both) for ProMisE molecular classification; detailed tumor pathology information, including hormonal receptor status; documentation of any treatment received; and clinical outcomes evaluated with at least six months of follow-up. The exclusion criteria were as follows: molecular profiles different from NSMP, incomplete molecular profiling, adjuvant treatment executed at other institutions, fertility-sparing surgery, ECOG score >3, and synchronous endometrial and ovarian carcinomas.

All surgical interventions were performed in accordance with European guidelines for the management of patients with endometrial carcinoma, ensuring standardized and evidence-based practices across all cases [19]. The standard surgical treatment was laparoscopic hysterectomy and bilateral salpingo-oophorectomy with lymph node staging (SLN assessment using indocyanine green or pelvic lymphadenectomy for low-grade cases, and pelvic and para-aortic lymphadenectomy for high-grade and/or non-endometrioid histology); in advanced stages, debulking surgery was performed to remove all visible disease.

### 2.2. Data Collection

Patient data were collected prospectively and anonymized to ensure confidentiality. A dedicated database was created for this purpose, compliant with data protection regulations. Each patient was assigned a unique identification code, and all personal identifiers were removed before data entry. The anonymized data were stored securely and were accessible only to authorized research personnel.

For each patient, detailed clinical and pathological characteristics were collected, including patient demographics, such as age, body mass index (BMI), menopausal status, and comorbidities; surgical details, including type of surgery performed, type of lymph node assessment and intraoperative findings; post-surgical adjuvant therapy; pathological characteristics, namely histological type, tumor grade, lymph vascular space invasion (LVSI) (descripted as absent, focal or substantial), myometrial invasion, cervical or vaginal involvement, lymph node status, tumor stage according to FIGO 2009 classification system [24] and the risk class assessment according to the aforementioned guidelines [19]. 

All patients were monitored through our department’s routine oncologic follow-up program, which involved four clinical examinations per year for the first 2 years, three annual examinations until the fourth year, and then two visits per year thereafter. Each follow-up included an ultrasound examination, while radiologic imaging was performed every 12 months or in the case of suspected relapse. Only patients with at least a 6-month follow-up were included in the statistical analysis.

To ensure the accuracy and completeness of the data, regular audits were conducted. Any discrepancies identified were resolved by cross-referencing the original medical records. The integrity of the database was maintained through stringent data entry protocols and routine validation checks.

### 2.3. Outcomes of the Study

The primary outcome of our study was to evaluate if dividing NSMP ECs into two risk subgroups (low-risk and high-risk) based on ER status, histotype, and grade, according to the previously proposed model [23], can more accurately predict prognosis. NSMP tumors were classified as low-risk (LR-NSMP) if they exhibited both endometrioid histotype, low grade (G1–G2), and ER positivity, expressing the ‘ER-positive/endometrioid/low-grade’ phenotype. Conversely, NSMP ECs with alterations in any one of these three factors were classified as high-risk NSMP (HR-NSMP).

Secondary outcomes included the following: stratifying NSMP prognoses in relation to ER status and comparing these with the previously proposed model and the FIGO staging system; and defining the proportion of NSMPs that would likely require escalation or de-escalation of adjuvant treatment.

### 2.4. Immunohistochemistry and Next Generation Sequencing

According to international guidelines and recent publications [19,25,26], endometrial tumor tissues were collected during pre-operative biopsies and surgical procedures, and formalin-fixed and paraffin-embedded (FFPE) for the immunohistochemical (IHC) molecular characterization. This process involved the analysis of the MMR status using an IHC antibody panel for the evaluation of the expression of four proteins (PMS2, MLH1, MSH2 and MSH6): loss of expression of any MMR protein was indicative of MMR deficiency. Also, the analysis of p53 in EC was performed by an immunohistochemical antibody panel, where aberrant p53 expression patterns included complete absence of staining (null pattern) or strong, diffuse nuclear staining in >50% of tumor cells (overexpression pattern), indicative of *TP53* mutation. *POLE* mutational status was assessed by next-generation sequencing using the Myriapod^®^ NGS Cancer panel DNA. POLE exonuclease domain mutations (EDMs) were considered pathogenic according to defined criteria [27]. The presence or absence of tumor expression of estrogen receptor (ER) and progesterone receptor (PR) were also routinely evaluated using immunohistochemical assays. ER and PR immunoreactivity was scored based on the percentage of positive tumor cell nuclei and the intensity of staining. A cut-off value of ≥1% positive cells was used to define ER positivity.

### 2.5. Statistical Analysis

Statistical analyses were performed using MedCalc for Windows, version 19.4 (MedCalc Software, Ostend, Belgium). Patient data were analyzed by descriptive statistics. Continuous variables were summarized as median with interquartile range (IQR) and analyzed by the Kruskal–Wallis test. Categorical variables were expressed as a percentage and analyzed using the chi-square (χ2) test or Fisher’s test when appropriate. Survival analyses were performed for the endpoints of disease-free survival and overall survival using log-rank test and visualized through Kaplan–Meier curves. Disease-free survival (DFS) was defined as the time from the date of surgery to the date of the first documented recurrence (local or distant) or death from any cause; overall survival (OS) was defined as the time from the date of surgery to the date of death from any cause. Patients without any recurrence or death were censored at the last follow-up date. Statistically significant differences were defined as a *p*-value < 0.05.

## 3. Results

### 3.1. Study Population and Histopathological Features

**Figure 1:**
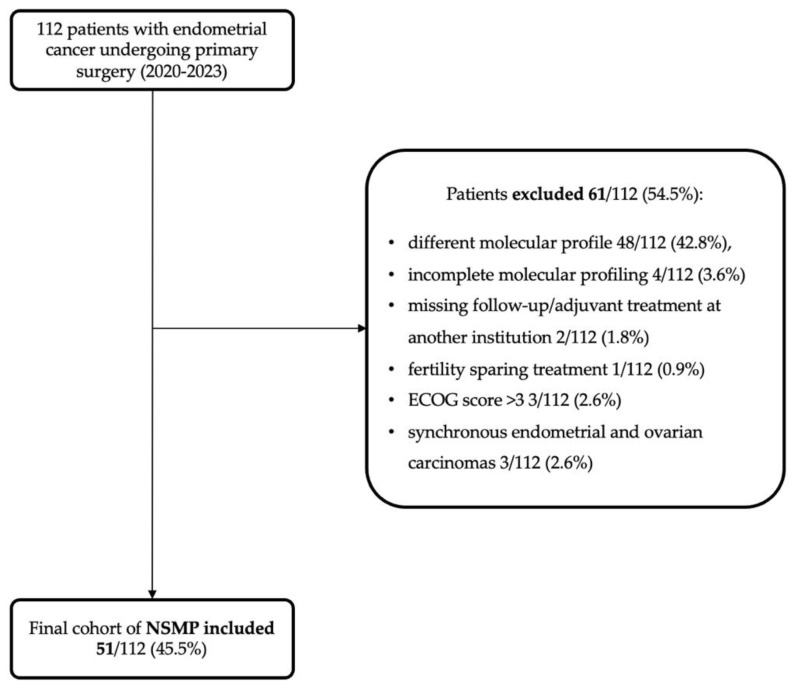
Flowchart showing the application of exclusion criteria to arrive at the study cohort (51 NSMPs).

During the study period, 112 endometrial carcinomas (ECs) were diagnosed, managed, and treated. Of these, after applying exclusion criteria (Figure 1), 51 (45.5%) were classified as NSMP, forming the study group.

Table 1 provides a comprehensive description of clinical and histopathological characteristics of the NSMP cohort. The median age at the time of treatment was 64 years (IQR 56.5–75), with a median BMI of 28 kg/m^2^ (IQR 24–33). More than one-third of the patients (35.2%) were obese, and within this group, nearly 59% had grade II obesity, defined as BMI > 35 kg/m^2^. All patients successfully underwent primary surgery without residual disease. Median follow-up within the subgroup of NSMP ECs was 16.0 (IQR 13.0–29.8) months.

Forty-four tumors were classified as endometrioid carcinomas (86.3%), and seven were of other histologies (13.7%), specifically one serous carcinoma (2%), two clear-cell carcinomas (3.9%), one carcinosarcoma (2%), two mesonephric-like carcinomas (3.9%), and one dedifferentiated carcinoma (2%). Most NSMP EC (78.4%) patients were classified as low grade (grades 1 and 2). Nearly all patients, 49 out of 51 NSMP ECs, exhibited some degree of myometrial invasion, and 24 had invasion of less than 50%. According to the 2009 FIGO staging system, the histopathological stage at diagnosis was IA in 24 patients (47.1%), IB in 14 patients (27.5%), and higher stages in the remaining 13 patients (25.4%), with lymph node positivity, resulting in a stage IIIC, observed in five patients (9.8%). With regard to the adjuvant treatment performed, 25 patients (49%) did not require any adjuvant therapy and were assigned to follow-up, 11 (21.6%) received vaginal brachytherapy (VBRT) only, 7 (13.7%) had external beam radiotherapy (EBRT) ± VBRT, and the remaining 8 (15.5%) received both chemotherapy and radiotherapy (CTRT) with or without VBRT.

**Figure 2:**
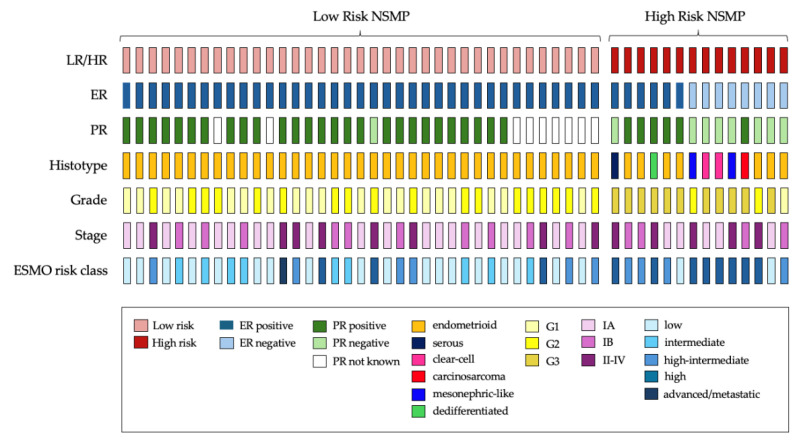
Oncoplot summarizing clinicopathological and immunohistochemical features, and risk classification of NSMP ECs cohort. LR, low-risk NSMP; HR, high-risk NSMP; ER, estrogen receptor; PR, progesterone receptor; ESMO, European Society of Medical Oncology.

The majority of NSMP tumors demonstrated ER and PR positivity, with only three cases expressing positivity for only one of the two receptors (see Figure 2 for further details). Loss of ER expression was observed in eight NSMP ECs (15.7%), while PR loss was noted in nine cases (21.4%). PR status was not available in nine histopathological reports. A descriptive statistical analysis comparing ER-positive and ER-negative tumors is provided in the Supplementary Materials (Table S1).

### 3.2. Low-Risk (LR) vs. High-Risk (HR) NSMPs

We stratified the NSMP cohort based on ER status, grade, and histotype. Patients with ‘ER-positive/endometrioid/low-grade’ phenotype formed the low-risk group (LR-NSMP). All other tumors were classified as HR-NSMPs. Using this stratification, we identified 37 LR-NSMP (72.5%) and 14 HR-NSMP (27.5%). Table 1 details the patients’ and histology characteristics, the outcomes of the two subgroups, and the comparison. Since no statistical difference in terms of FIGO stages was found between high and low risk (*p* = 0.514), we consider the study group homogenous. We reported a BMI significantly lower in HR-NSMP ECs compared to LR, with values of 24 kg/m^2^ vs. 29 kg/m^2^, respectively (*p* < 0.01). Notably, substantial LVSI was significantly more present in HR-NSMPs than in LR-NSMPs, with 21.4% vs. 13.5%, respectively (*p* < 0.042).

A visual representation of individual cases is provided in Figure 2. Within the HR-NSMP group, 7 out of 14 tumors (50%) exhibited a non-endometrioid histotype, while only 3 out of 14 (21.4%) were classified as low grade. Estrogen receptor loss was observed in eight cases (57.1%), whereas six cases (42.9%) retained estrogen receptor positivity.

According to the ESMO (European Society for Medical Oncology) 2023 risk stratification systems (Table 2), most of our cases (39.2%) were low-risk ECs, while 21.6% were identified as high risk. As expected, a higher proportion of high-risk ECs was found in the HR-NSMP group (57.1% vs. 8.1%), whereas a greater proportion of low-risk ECs was observed in the LR-NSMP group (48.7% vs. 14.3%, *p* < 0.008). Notably, in our study population, only one case was diagnosed as advanced/metastatic, with cancer localized in the fibula. Surprisingly, this case belonged to the LR-NSMP group and was successfully treated with stereotactic body radiotherapy. The patient did not report any recurrence of disease during follow-up. Among HR-NSMPs, three tumors expressed the ‘Endometrioid/low-grade/ER-negative’ phenotype, with two diagnosed as localized disease (IA-IB) and one as advanced stage (II-IV). Using the ESMO risk classification, approximately 14% (2/14) of ECs in the HR-NSMP group would be classified as low and intermediate risk, respectively, while all others were classified as high–intermediate to high risk.

### 3.3. Survival Analysis

**Figure 3:**
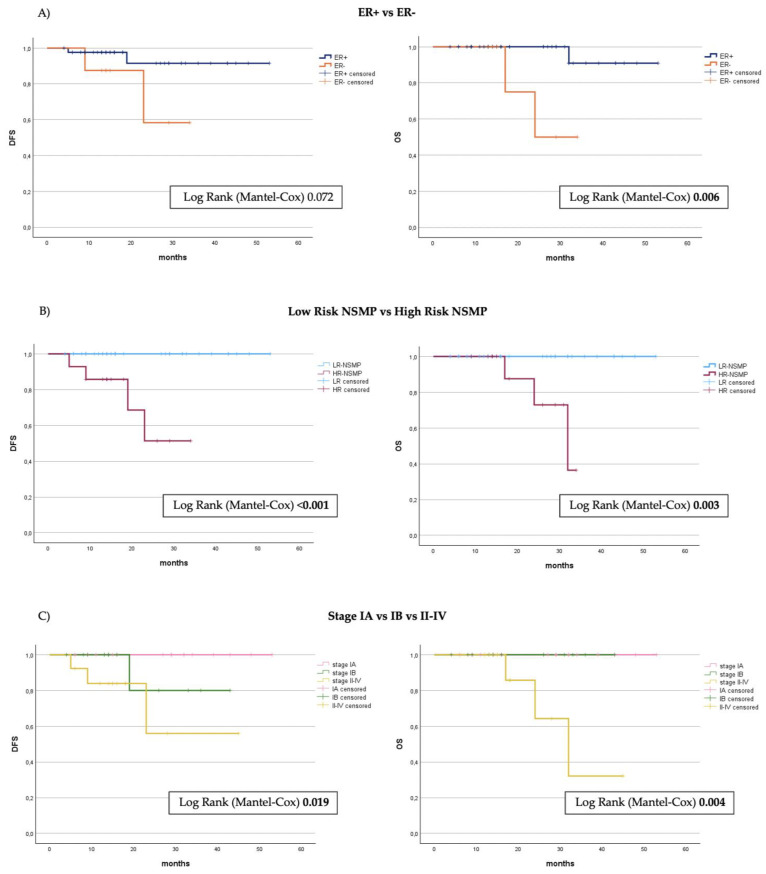
Kaplan–Meier curves showing disease-free survival (PFS) and overall survival (OS) from the timepoint of primary surgery stratified by (**A**) ER status, (**B**) division into risk groups, (**C**) FIGO stage 2009. ER, estrogen receptor; NSMP, no specific molecule profile.

Kaplan–Meier curves and the log-rank test were applied to highlight differences in terms of OS and DFS. All cases with disease recurrence and those resulting in death occurred in the high-risk NSMP group (*p* < 0.001 and *p* = 0.004, respectively). There were no such cases in the low-risk group. Four out of fourteen cases (28.6%) experienced recurrence and were treated with platinum-based chemotherapy, and two cases were treated with secondary cytoreductive surgery. Three of these patients (21.4%) subsequently died due to the disease, all of whom initially presented with FIGO stage higher than I. Figure 3 shows the survival curves of the two subgroups, low-risk and high-risk, which are significantly different in terms of both DFS (*p* < 0.001) and OS (*p* = 0.003). At a mean follow-up of 22 months in the HR-NSMP group, the DFS is approximately 70% and the OS is 85.7%, compared to 100% in both metrics for the low-risk subgroup. We conducted a specific analysis to evaluate the role of ER. Two cases of relapse occurred in the ER-positive carcinomas and two in the ER-negative group. Kaplan–Meier curves indicated no significant difference in disease recurrence between the two groups (*p* = 0.072). In contrast, ER-positive NSMPs showed significantly improved cumulative survival (*p* = 0.006), which was more consistent with the results observed when dividing cases into the two risk classes.

## 4. Discussion

The present study attempted to stratify NSMP subtype endometrial carcinomas and better define their prognoses. Indeed, they represent the most common molecular subtype, accounting for almost 50% of ECs [1], and a highly heterogeneous molecular class, essentially defined by excluding the other three classes (*POLE*mut, MMRd, and p53abn). As a result, survival curves for NSMP ECs, while generally intermediate between *POLE*mut and p53abn carcinomas, have shown considerable variability across different studies [2,3,4,5,6,7]. This study aims to evaluate prognostic outcomes obtained by the stratification within NSMP endometrial carcinomas into two risk classes, based on grade, histotype and estrogen receptor status, following the model proposed by a large multicenter Canadian study last year [23]. The division of NSMP tumors into low-risk and high-risk appears to define two different subgroups of ECs, with completely different disease-free survival and overall survival. NSMPs were classified as high-risk (HR) if they possessed at least one of the following features: ER-negative status, non-endometrioid histotype, or high grade (G3); the remaining cases were classified as low-risk (LR-NSMP). In our sample, HR-NSMP accounted for 27.5% of NSMP tumors and were characterized by a significantly increased incidence of recurrence and disease-related deaths over the years, with four cases of recurrence, three of which resulted in death at a median follow-up of 16 months. The remaining NSMP patients, which constitute the majority, were classified as low-risk (LR-NSMP). A total of 37 LR-NSMP patients were identified, accounting for 72.5% of the sample, lower than the 84% of ECs found in the previous study on this model [23]. In this subgroup, no disease recurrence or related deaths were observed. From our perspective, these patients best represent the molecular NSMP EC prototype, characterized by endometrioid histotype, ER-positive status, and low grade (G1–G2), thus corresponding to the classical type I EC, associated with a very favorable prognosis.

Recently, as oncological treatment is increasingly tailored to specific molecular profiles to identify targets for therapies and to customize medical, surgical, and radiotherapy treatments, the management of endometrial carcinoma (EC) has undergone significant changes with the introduction of molecular classification. This classification into four molecular subgroups has demonstrated both prognostic and predictive value, guiding therapeutic decisions. The presence of pathogenic mutations in the *POLE* gene is associated with significantly better oncological outcomes, characterized by a very low risk of recurrence. Consequently, this has led the scientific community towards the de-escalation of adjuvant treatment, recommending follow-up alone for these patients. On the other hand, a p53abn profile defines a cluster of ECs with the highest risk of recurrence and the poorest prognosis, needing adjuvant radiotherapy ± concurrent chemotherapy in the presence of myometrial invasion [9]. NSMP and MMRd ECs have an intermediate prognosis, and their adjuvant treatment still primarily relies on traditional clinico-pathological characteristics such as stage, grade, and histotype. However, recent studies have highlighted a high mutational burden and a significant presence of tumor-infiltrating lymphocytes in MMRd ECs, leading to the approval of immune check-point inhibitors (ICIs) in advanced or recurrent disease settings [10]. The latest ESGO/ESTRO/ESP guidelines from late 2020 [19], along with the recent ESMO 2023 guidelines [18], have firstly proposed and then confirmed a risk group classification that incorporates molecular subgroups (Table 2). Furthermore, the new FIGO classification of 2023 has, for the first time in gynecological tumors, included a staging system based not only on disease extent but also on histopathological (such as histotype, grade, LVSI status) and molecular features, namely, *POLE* mutations, and p53 status [21]. The latter two result in down-staging and up-staging, respectively.

**Figure 4:**
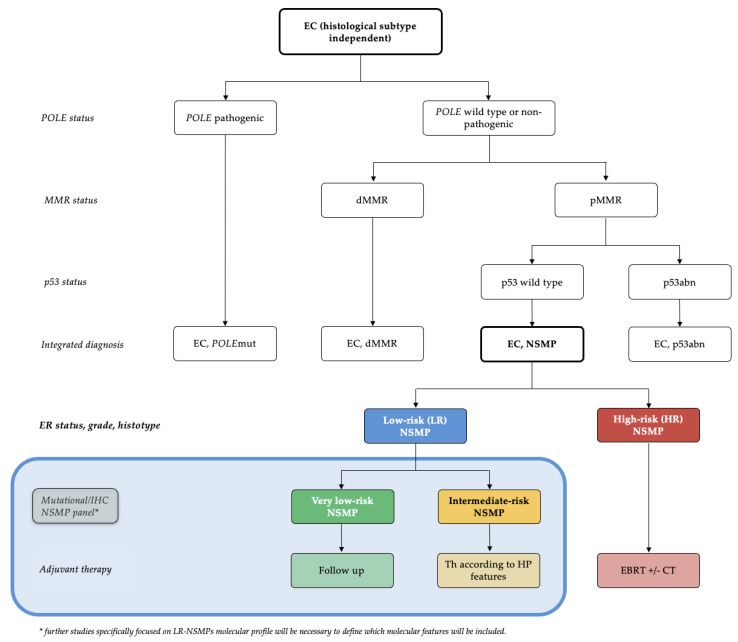
Diagnostic algorithm for the management of ECs: Based on the validated molecular classification, we define two risk groups obtained from NSMP ECs with completely different risks of recurrence and prognosis. The blue box presents a hypothetical future prospective derived from this study, leading to a molecular subclassification of LR-NSMP into very low-risk and intermediate-risk NSMPs. EC, endometrial carcinoma; dMMR, mismatch repair-deficient; NSMP, no specific molecular profile; *POLE*mut, POLE (DNA Polymerase Epsilon)-mutated/ultramutated; p53abn, p53-abnormal; HP, histopathological; EBRT, external beam radiation therapy; CT, chemotherapy.

Currently, the NSMP molecular group is poorly defined. It is considered a “big box” that includes all patients without specific molecular features. In light of the results of this study and previous research [23], we believe that grouping NSMPs into high-risk and low-risk groups in clinical research could contribute to obtaining more homogeneous samples with consistent prognoses, avoiding selection bias arising from NSMP heterogeneity. Future applications of this subdivision in clinical practice require further validation of this model, but we have attempted to hypothesize these applications in the subsequent sections and summarize them in a management flowchart (Figure 4).

### 4.1. Potential Implications for Management of High Risk-NSMP

Within this group, three subgroups of neoplasms were identified: (1) G3 endometrioid carcinomas, considered to be at high–intermediate risk of recurrence according to guidelines [18], requiring adjuvant radiation ± concurrent chemotherapy (EBRT ± ChT); (2) non-endometrioid ECs, known for their high risk of recurrence, requiring post-surgical concurrent radio-chemotherapy or systemic chemotherapy; and (3) low-grade ER-negative endometrioid carcinomas. The inclusion of this third subgroup is based on the 2023 study by Lisa Vermij et al., which demonstrated that ER negativity in this population is a very strong independent negative prognostic factor, and it is confirmed by our study, although on its own, that it demonstrates a lower strength compared to the subdivision into risk groups. In our cohort, as illustrated in Figure 2, two patients fell into the HR-NSMP category without expressing either G3 or a non-endometrioid histotype, characterized by the phenotype “Endometrioid/low-grade/ER-negative”. Tumors with this phenotype, although rare (representing approximately 3–4% of endometrial tumors, 3.9% in our cohort, 4 cases out of 161—2.5% in the cohort of Vermij et al. [22]), could also benefit from adjuvant treatment with EBRT±ChT. Indeed, endometrial carcinomas that are ER-negative display a more aggressive clinicopathologic phenotype and are likely distinguished from ER-positive tumors by distinct genetic alterations and gene expression profiles. Molecular studies of mRNA associated with ER loss and epithelial–mesenchymal transition (EMT)-related transcription factors have demonstrated a correlation between low ER levels and increased expression of EMT factors. EMT enables epithelial cells to acquire mesenchymal traits, enhancing their motility, local invasion, intravasation, circulation, extravasation, and resistance to apoptosis [28].

### 4.2. Potential Implications for Management of Low-Risk NSMP

As mentioned earlier, NSMP endometrial carcinomas require further investigation to better define their molecular alterations and prognostic characteristics. Currently, few studies have focused on selectively investigating the molecular patterns of NSMP tumors, and none have specifically isolated this low-risk population. However, it is precisely this population that remains poorly understood and warrants more attention, particularly in the context of a de-escalation strategy. Initial efforts in this direction were made by Jamieson et al., who, among the secondary outcomes of their study, evaluated the association between IHC alterations and mutations in genes frequently associated with more aggressive behavior and a higher risk of recurrence in other pathologies [23]. Additionally, a recent study by Onoprienko et al. examined the prognostic role of ARID1A in patients with NSMP EC [29]. The former study identified only L1CAM overexpression (IHC) and PI3KCA mutation as factors associated with a higher risk of recurrence (with respective HRs of 4.07, 95% CI 2.06–7.66, *p* = 0.0002, and HR 3.3, 95% CI 1.21–10.04, *p* = 0.0188). An earlier study also demonstrated that L1CAM status was predictive of worse outcomes among NSMP subtype ECs, with an HR of 7.82 (*p* < 0.001) [14]. Other molecular features investigated, such as ARID1A expression, CTNNB1 mutation, and KRAS mutation, were not significantly associated with an increased risk. Conversely, Onoprienko et al.’s study [29] identified ARID1A as a risk factor for recurrence (HR 3.96, 95% CI 1.41 to 11.15, *p* = 0.009) but did not associate it with an increase in disease-specific survival in their cohort. Similarly, an association between ARID1A expression and poor prognosis in NSMPs was also previously found by De Leo et al. [30].

A new diagnostic algorithm that accounts for the subdivision of NSMP ECs into low-risk and high-risk categories can be found in Figure 4. The blue box illustrates what we believe could be a future development in the management of LR-NSMP, focusing on a more personalized treatment approach: the creation of a mutational panel assessable by IHC and next-generation sequencing (NGS). This panel should consider the results of recent studies and include L1CAM, PI3KCA, ARID1A and potentially other markers such as the amplification of 1q32.1 [16]. This panel could further define an intermediate-risk group, characterized by the presence of factors that worsen prognosis, and a true low-risk group, termed very-low-risk, which would be directed to follow-up within a de-escalation strategy framework. With this approach, we could significantly increase the number of endometrial cancers managed with follow-up alone, in addition to *POLE*mut tumors, which currently represent only 5–8% of ECs.

### 4.3. Strengths and Weaknesses of This Study

The current study represents the first external application of the subdivision of NSMP ECs into risk classes, according to the model proposed by Jamieson et al. [23] Data were prospectively collected as part of a broader study protocol on the application of molecular classification to endometrial carcinomas since its introduction at our center; therefore, the follow-up is regular and standardized. This study highlights the difference in survival outcomes between the two subgroups of NSMP. Another important factor is the short time interval in which the patients were treated: during this period, the guidelines for adjuvant treatment did not change, ensuring uniformity in patient treatment. This avoids a significant bias commonly found in studies with a long enrollment period.

The limitations of this study include the following: (1) the retrospective nature of the analysis, despite the data being collected prospectively, may lead to incomplete data collection and potential non-uniformity between the two groups; (2) the results should currently be interpreted with caution due to the limited number of patients and follow-up; (3) the absence of recurrence cases in low-risk tumors due to the generally good prognosis of the disease; and (4) the absence of a validated ER positivity cutoff in endometrial cancer. Nevertheless, we believe that the validation of this model is of interest to the scientific community, as its application in future studies could lead to more homogeneous populations in terms of survival outcomes compared to the entire cohort of NSMP ECs.

## 5. Conclusions

In conclusion, our study highlights the existence of two subgroups with markedly different prognoses within the heterogeneous landscape of the no specific molecular profile (NSMP) subtype of endometrial carcinomas. This subdivision, if confirmed, could lay the groundwork for a significant shift in clinical and therapeutic approaches and may also be important for standardizing NSMP cohorts in future studies on this topic.

## Figures and Tables

**Table 1:** Descriptive statistics of clinicopathologic features and oncological events of NSMP full cohort and divided into the two risk classes.

	Total Cohort	Low-Risk	High-Risk	*p * ^1^
**Total**	51 (100)	37 (72.5)	14 (27.5)	
**Age** **(y), median (IQR)**	64 (56.5–75)	67 (57.5–73.8)	61 (56–73)	0.597
**Age, categorized**				
≤60	19 (37.3)	12 (32.4)	7 (50.0)	0.251
>60	32 (62.7)	25 (67.6)	7 (50.0)	
**BMI** **(kg/m^2^** **), median (IQR)**	28 (24–33)	29 (25.7–36)	24 (23–25)	**0.001**
**Type of Surgery**				
Nodal assessment	44 (86.3)	32 (86.5)	12 (85.7)	0.470
No nodal assessment	7 (13.7)	5 (13.5)	2 (14.3)	
**Grade**				
Low Grade (G1–G2)	40 (78.4)	37 (100)	3 (21.4)	**<0.001**
High Grade (G3)	11 (21.6)	0 (0)	11 (78.6)	
**Histological Type**				
Endometrioid	44 (86.3)	37 (100)	7 (50)	**<0.001**
Non endometrioid	7 (13.7)	0 (0)	7 (50)	
**LVSI**				
Absent	41 (80.4)	32 (86.5)	9 (64.3)	
Focal	2 (3.9)	0 (0)	2 (14.3)	**0.042**
Substantial	8 (15.7)	5 (13.5)	3 (21.4)	
**Myometrial Invasion**				
Absent	2 (3.9)	2 (5.4)	0 (0)	0.379
Present	49 (96.1)	35 (94.6)	14 (100)	
**Cervical Invasion**				
Absent	43 (84.3)	31 (83.8)	12 (85.7)	0.867
Present	8 (15.7)	6 (16.2)	2 (14.3)	
**Vaginal Invasion**				
Absent	49 (96.1)	36 (97.3)	13 (92.8)	0.470
Present	2 (3.9)	1 (2.7)	1 (7.2)	
**ER status**				
Positive	43 (84.3)	37 (100)	6 (42.9)	**<0.001**
Negative	8 (15.7)	0 (0)	8 (57.1)	
**PR status**				
Positive	33 (78.6)	27 (96.4)	6 (42.9)	
Negative	9 (21.4)	1 (3.6)	8 (57.1)	**<0.001**
Unknown				
**FIGO Stage 2009**				
IA	24 (47.0)	19 (51.4)	5 (35.7)	
IB	14 (27.5)	10 (27)	4 (28.6)	0.514
II-IV	13 (25.5)	8 (21.6)	5 (35.7)	
**Risk Class** **(ESGO/ESTRO/ESP 2020)**				
Low	20 (39.2)	18 (48.7)	2 (14.3)	
Intermediate	9 (17.6)	9 (24.3)	0 (0)	
Intermediate–High	10 (19.6)	6 (16.2)	4 (28.6)	**<0.001**
High	11 (21.6)	3 (8.1)	8 (57.1)	
Advanced	1 (2)	1 (2.7)	0 (0)	
**Adjuvant Treatment**				
None	25 (49.0)	21 (56.7)	4 (28.5)	
VBRT only	11 (21.6)	9 (24.3)	2 (14.2)	
EBRT ± VBRT	7 (13.7)	5 (13.5)	2 (14.2)	**0.010**
CTRT ± VBRT	8 (15.5)	2 (5.4)	6 (42.8)	
CT only	0	0	0	
**Survival**				
Recurrence	4 (7.8)	0 (0)	4 (28.6)	**<0.001**
Died of Disease	3 (5.9)	0 (0)	3 (21.4)	**0.004**

Values are given as *n* (%) unless otherwise specified. BMI, body mass index; LVSI, lymphovascular space invasion; ER, estrogen receptor; PR, progesterone receptor; EBRT, external beam radiation therapy; VBRT, vaginal brachytherapy; CTRT, chemo-radiotherapy. FIGO, International Federation of Gynaecology and Obstetrics; ESGO, European Society of Gynaecological Oncology; ESTRO, European Society for Radiotherapy & Oncology; ESP, European Society of Pathology. ^1^ *p* values < 0.05 were considered significant.

**Table 2:** ESMO 2023 risk groups [18].

Risk Group	Description
Low risk	Stage IA (G1–G2) with endometrioid type (dMMR and NSMP) and no or focal LVSI
	Stage I/II *POLE*mut cancer; for stage III *POLE*mut cancers
Intermediate	Stage IA G3 with endometrioid type (dMMR and NSMP) and no or focal LVSI
	Stage IA non-endometrioid type (serous, clear-cell, undifferentiated carcinoma, carcinosarcoma, mixed) and/or p53-abn cancers without myometrial invasion and no or focal LVSI
	Stage IB (G1–G2) with endometrioid type (dMMR and NSMP) and no or focal LVSI
	Stage II G1 endometrioid type (dMMR and NSMP) and no or focal LVSI
High-intermediate	High–intermediate risk Stage I endometrioid type (dMMR and NSMP) any grade and any depth of invasion with substantial LVSI
	Stage IB G3 with endometrioid type (dMMR and NSMP) regardless of LVSI
	Stage II G1 endometrioid type (dMMR and NSMP) with substantial LVSI
	Stage II G2–G3 endometrioid type (dMMR and NSMP)
High risk	All stages and all histologies with p53-abn and myometrial invasion
	All stages with serous or undifferentiated carcinoma including carcinosarcoma with myometrial invasion
	All stage III and IVA with no residual tumor, regardless of histology and regardless of molecular subtype

dMMR, mismatch repair-deficient; NSMP, no specific molecular profile; LVSI, lymphovascular space invasion; *POLE*mut, POLE (DNA Polymerase Epsilon)-mutated/ultramutated; p53-abn, p53-abnormal.

## Data Availability

The data presented in this study are available upon request from the authors.

## Supplementary Materials

The following supporting information can be downloaded at: https://www.mdpi.com/article/10.3390/cancers16183221/s1, Table S1: Descriptive statistics of clinicopathologic features and oncological events of NSMP full cohort and divided into ER-positive and ER-negative tumors.
