# Supplementary material for: Low-Risk and High-Risk NSMPs: A Prognostic Subclassification of No Specific Molecular Profile Subtype of Endometrial Carcinomas
Source: Cancers (Basel). 2024 Sep 21;16(18):3221. doi: 10.3390/cancers16183221 (PMC11429616; doi:10.3390/cancers16183221)
Supplement: Supplementary file 1 [file cancers-16-03221-s001.zip › cancers-3132997-supplementary.pdf]

Supplementary Material

Tables

Table S1. Descriptive statistics of clinicopathologic features and oncological events of NSMP full cohort and divided into ER-positive and ER-negative tumors.

	Total Cohort	ER +	ER -	p^1
**Total**	51 (100)	43 (84.3)	8 (15.7)	
**Age (y), median (range)**	64 (56.5-75)	67 (58-73)	58.5 (54.5-71)	0.357
**Age, categorized**				
≤ 60	19 (37.3)	14 (32.5)	5 (62.5)	0.111
> 60	32 (62.7)	29 (67.5)	3 (37.5)	
**BMI (kg/m2), median (range)**	28 (24-33)	29 (24-35.5)	24.5 (22-25.5)	**0.021**
**Type of Surgery**				
Nodal assessment	44 (86.3)	37 (86.0)	7 (87.5)	0.930
No nodal assessment	7 (13.7)	6 (14.0)	1 (12.5)	
**Grade**				
Low Grade (G1-G2)	40 (78.4)	37 (86.0)	3 (37.5)	**0.008**
High Grade (G3)	11 (21.6)	6 (14.0)	5 (62.5)	
**Histological Type**				
Endometrioid	44 (86.3)	41 (95.3)	3 (37.5)	**<0.001**
Non endometrioid	7 (13.7)	2 (4.7)	5 (62.5)	
**LVSI**				
Absent	41 (80.4)	35 (81.3)	6 (75.0)	
Focal	2 (3.9)	1 (2.4)	1 (12.5)	0.391
Substantial	8 (15.7)	7 (16.3)	1 (12.5)	
**Myometrial Invasion**				
Absent	2 (3.9)	2 (4.7)	0 (0)	0.537
Present	49 (96.1)	41 (95.3)	8 (100)	
**Cervical Invasion**				
Absent	43 (84.3)	36 (83.7)	7 (87.5)	0.789
Present	8 (15.7)	7 (16.3)	1 (12.5)	
**Vaginal Invasion**				
Absent	49 (96.1)	42 (97.6)	7 (87.5)	0.177
Present	2 (3.9)	1 (2.4)	1 (12.5)	
**NSMP risk class**				
Low-risk	43 (84.3)	37 (86.0)	0 (0)	**<0.001**
High-risk	8 (15.7)	6 (14.0)	8 (100)	
**PR status**				
Positive	33 (78.6)	32 (74.4)	1 (12.5)	
Negative	9 (21.4)	2 (4.6)	7 (87.5)	**<0.001**
Unknown	9 (21.4)	9 (21.0)	0 (0)	
**FIGO Stage 2009**				
IA	24 (47.0)	21 (48.8)	3 (37.5)	
IB	14 (27.5)	12 (27.9)	2 (25.0)	0.690
II-IV	13 (25.5)	10 (23.3)	3 (37.5)	
**Risk Class (ESGO/ESTRO/ESP 2020)**				
Low	20 (39.2)	19 (44.1)	1 (12.5)	

Intermediate	9 (17.6)	9 (20.9)	0 (0)	
Intermediate–High	10 (19.6)	9 (20.9)	1 (12.5)	**0.002**
High	11 (21.6)	5 (11.7)	6 (75.0)	
Advanced	1 (2)	1 (2.4)	0 (0)	
**Adjuvant Treatment**				
None	25 (49.0)	23 (53.5)	2 (25.0)	
VBRT only	11 (21.6)	9 (20.9)	2 (25.0)	
EBRT+/-VBRT	7 (13.7)	7 (16.3)	0 (0)	**0.022**
CTRT +/- VBRT	8 (15.5)	4 (9.3)	4 (50.0)	
CT only	0	0	0	
**Survival**				
Recurrence	4 (7.8)	2 (4.6)	2 (25.0)	0.052
Died of Disease	3 (5.9)	1 (2.3)	2 (25.0)	**0.013**

Values are given as n (%) unless otherwise specified.
BMI, body mass index; LVSI, lymphovascular space invasion; ER, estrogen receptor; PR, progesterone receptor; EBRT, external beam radiation therapy; VBRT, vaginal brachytherapy; CTRT, chemo-radiotherapy.
FIGO, International Federation of Gynaecology and Obstetrics; ESGO, European Society of Gynaecological Oncology; ESTRO, European Society for Radiotherapy & Oncology; ESP, European Society of Pathology.
[1] p values <.05 were considered significant.
